# Challenges encountered to creating a renal biopsy program at a tertiary care academic institution in the United States

**DOI:** 10.1080/0886022X.2024.2449576

**Published:** 2025-01-16

**Authors:** Eily Hayes, Erik Mai, Andre Uflacker, Natalie Freidin

**Affiliations:** a Division of Nephrology, Medical University of South Carolina, Charleston, SC, USA; b Vascular and Interventional Radiology, Savannah Vascular Institute, Savannah, GA, USA

**Keywords:** Native renal biopsy, nephrology fellowship, education, nephrology fellows, nephrology guided renal biopsy

## Abstract

Biopsy is the gold standard for diagnosing renal pathology and the procedure is required to be learned per ACGME guidelines for Nephrology Fellowship graduation. We describe the process for the planning and development of a new Nephrologist directed native renal biopsy program to increase the opportunity to train Nephrology fellows in this procedure. The article outlines the barriers, complications and lessons learned to developing the program, highlighting the key challenges and progress that has been made within a single American tertiary academic medical center.

## Background and introduction

Biopsy is the gold standard for diagnosing renal pathology [1]. The ACGME program requirements for graduate medical education in nephrology note that fellows should be provided with the opportunity to train and achieve competence in the performance of kidney biopsies [2]. Historically, performing renal biopsies was fundamental to the practice of nephrology. More recently and with the expansion of interventional radiology, this responsibility has shifted from nephrology to radiology [3, 4]; the same trend is evident at our institution. Department restructuring and COVID-19 pandemic-related staffing turnover significantly decreased fellows’ opportunity to perform kidney biopsies. To the uninitiated, creating a new training opportunity at an American tertiary care academic medical center might seem a straightforward task. We discuss the obstacles encountered in the development of a program for nephrology fellows to obtain training to perform percutaneous renal biopsies from other nephrologists, and strategies implemented to overcome these obstacles.

Imaging-guided renal biopsy – either CT-guided or with ultrasound – is becoming the standard of care due to similar or improved diagnostic yield and lower complication rates when compared to blind biopsies [5–7]. Compared to CT guidance, ultrasound is more widely available and less expensive to acquire and utilize; we thus focused our efforts exclusively on ultrasound guided renal biopsy training. Unless explicitly stated otherwise, all references to renal biopsy in our text refer to real-time ultrasound-guided percutaneous renal biopsies.

Tertiary care academic medical centers provide a wealth of training resources and exposure to a wide range of pathologies. The size and structure that make these centers ideal places to train also pose challenges to creating new training opportunities. Nephrology attendings and fellows encountered obstacles at nearly every step of the process: obtaining and storing the necessary supplies, establishing a safe and available location to perform biopsies, accessing trained ultra-sonographers and pathologists, and monitoring patient recovery, post-procedure. The nephrology department engaged stakeholders, found motivated attendings to champion the program, and, over the course of 5 years, overcame these obstacles to launch and expand a program that would allow them to train the next generation of nephrologists to perform native renal biopsies. Unanticipated benefits of the new biopsy program include shorter wait times for biopsy patients, improved biopsy sample cores obtained, and obtaining tissue diagnoses and initiating treatment earlier in the clinical course.

## Process and timeline

Throughout 2019, the Department of Vascular and Interventional Radiology (VIR) exclusively conducted all renal biopsies at our institution. Renal biopsy training for nephrology fellows was provided by interventionalists rather than nephrologists. An advantage to nephrology-performed renal biopsies is the use of a 16-gauge biopsy needle, compared to the 18-gauge biopsy needle favored by radiology. A recent study re-demonstrates that 16-gauge biopsy needles are significantly more likely to yield adequate tissue for diagnosis – with similar rates of complications – when compared to 18-gauge needles [8, 9].

Procuring renal biopsy needles presented one of the initial challenges faced at our facility. The hospital did not stock the needles, and there was no procurement process or purchasing protocol in place for the nephrology division to acquire them. Other supplies – including sterile gloves, local anesthetic and spinal needles for its injection, sterile ultrasound probe covers and gel, and sterile biopsy procedure kits (which contain sterile drapes, skin cleansing supplies, gauze 4 × 4 pads, and microscope slides) – were widely available and could be obtained from existing hospital supplies. However, the radiology department did not utilize automated 16-gauge biopsy needles. The nephrology faculty struggled to address supply acquisition with both the department business manager and the hospital’s central supplies, but budgetary constraints and resource limitations posed significant barriers. Finally, in the first quarter of 2020, the initial stock of automated biopsy needles was acquired from a nearby hospital, outside of our academic medical center.

At the start of the second quarter of 2020, nephrology faculty initiated collaboration with the radiology department. The two departments built rapport over the course of several meetings, agreed that fellows need to acquire proficiency in this gold-standard diagnostic skill, and implemented a shared training approach: nephrologists would provide training and supervision of fellows, and radiology would provide the use of ultrasound machines, trained ultra-sonographers, and facilities and staff for pre-biopsy patient preparation, performing biopsies, and post-biopsy recovery and monitoring. This approach was utilized successfully from 2020 through the third quarter of 2021. Unfortunately, retirement of the head of the radiology department, concurrent COVID-19 pandemic and related turnover of staff, and subsequent radiology department restructuring halted all ultrasound-guided diagnostic procedures. As a result, all renal biopsies shifted back to VIR.

Following this, nephrology sought to establish collaboration with VIR to revive the training program previously crafted by both Nephrology and Radiology. However, the increased workload rendered VIR unable to continue their support of nephrology fellow training.

In the first half of 2022, nephrology attendings spearheaded a stakeholder meeting, which included the VIR and surgery departments, nephrology department, transplant integrated centers of clinical excellence (ICCE), radiology department, and the CEO and business administrators of the hospital organization. It was suggested that ultrasound-guided native renal biopsies could perhaps be transitioned to the outpatient nephrology clinic, but discussions stagnated after it was pointed out that the clinic lacked beds for biopsy patients, nursing staff, and facilities for pre- and post-procedure monitoring, and support staff for possible post-biopsy complications. Unfortunately, the process again stalled, as did biopsies performed by nephrology fellows under the tutelage of nephrologists.

In June 2023, one of the nephrology associate program directors obtained certification in renal ultrasonography. The aim of certification was to decrease the need for additional staffing for renal biopsies while creating an opportunity for ultrasonography training for nephrology fellows and faculty with the broader aim of extending the use of ultrasonography for real-time assessment of renal anatomy and related risks and benefits of US-guided biopsy. Utilizing an ultrasound machine borrowed from the transplant nephrology service, biopsy training for fellows was reinstated. All biopsy patients would have CT or ultrasound renal imaging in the 6 weeks prior to the procedure, and meet our criteria for low-risk biopsy (see Table 1). The initial protocol involved admitting biopsy patients on the morning of the scheduled biopsy procedure under observation status. Patients would remain in the hospital until post- biopsy monitoring – including post-procedure ultrasound and serial monitoring of vital signs hemoglobin levels to evaluate for serious bleeding complications – to assess their safety for discharge. However, bed availability proved too unpredictable, and delayed admissions. As biopsies require the nephrology attending/ultrasonographer, the fellow, and the pathologist all to be present at bedside during the procedure, delays in admission created significant scheduling complications and resultant delayed or canceled biopsies. The biopsy protocol underwent another revision, with patients admitted the evening before their scheduled biopsy.

**Table 1.** Characteristics of low-risk biopsy patients.

BMI <30–35 Age <65 years Controlled BP < 140/90 No known coagulopathy or thrombocytopenia Off antiplatelet therapy for 10 days or DOACs for 5 days No structural kidney disease (including assessment for renal vein thrombosis with nephrotic syndrome) on imaging obtained within 6 weeks prior to the procedure

**Figure 1.**
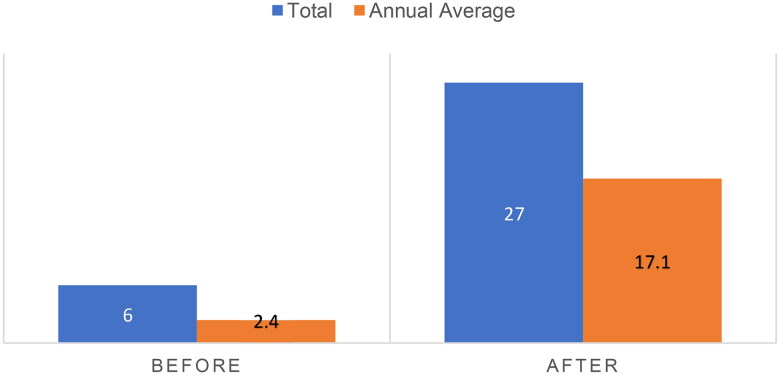
Renal biopsies are performed by nephrology fellows before and after the implementation of a protocol for the nephrology department to perform low-risk percutaneous native biopsies at the bedside.

The revised protocol was successful: the number of biopsies performed by nephrology fellows and supervised by nephrologists increased exponentially (see Figure 1). However, there were still obstacles to overcome: biopsies were dependent on the schedule of the sole ultrasound-trained nephrologist, and – without a dedicated procedure room – acquisition and storage of biopsy supplies remained problematic. The purchasing problem for 16-gauge automated biopsy needles was resolved, but they were stored in nephrology faculty offices. Biopsy needle guides – not required but helpful for obtaining biopsy samples – remained elusive until the latter half of 2023 despite the pursuit of multiple avenues to procure them. Other biopsy supplies had to be gathered from multiple storage locations throughout the hospital.

As the biopsy program evolved, the general nephrology and transplant nephrology programs collaborated to create a renal biopsy simulation lab program, pairing high-fidelity procedure training with a didactics curriculum based on *Kerns Curriculum Development for Medical Education*. Dedicated simulation lab training time was scheduled early in the fellowship to provide an introduction to ultrasound-guided biopsy, develop familiarity with ultrasound imaging of renal anatomy and muscle memory for the biopsy technique, and practice native and transplant biopsy techniques on mannequins.

The notable rise in native renal biopsies conducted by nephrology fellows under the new protocol spurred the progression of the process. This was due in part to the significantly higher costs to the institution associated with overnight patient admissions in comparison to same-day admission and discharge for the procedure. The intuition’s chief medical officer (CMO) reached out to nephrology, and subsequently became a proponent of the program to train nephrology fellows. The CMO’s involvement and efforts helped to motivate other stakeholders, and after a series of meetings, a procedure room was found in radiology that was being utilized only for training. This room became the new procedure room for the hospital’s nephrology biopsy clinic, and funding was made available for nursing staff for outpatient biopsy procedures, and for pre-biopsy preparation and post-biopsy recovery and monitoring. The anticipated launch of the new biopsy clinic in August 2023 was delayed because of setbacks in implementing electronic medical record (EMR) templates for scheduling and billing purposes. These delays were attributed to the hospital’s expansion and acquisition of a satellite hospital, demanding the attention of the available EMR software technicians.

In April 2024 – nearly 5 years after the conception of the initiative for nephrologists to train fellows in performing percutaneous native renal biopsies – the first biopsy was performed in the new nephrology outpatient biopsy clinic. With the new biopsy clinic came a procedure for purchasing all procedural supplies, including biopsy needles and kits, needle guides, sterile gloves, sterile ultrasound gel and probe covers, spinal needles, and biopsy needle guides, now all stored in the new clinic space.

From 2020–2024, 47 native renal biopsies were performed by fellows under direct supervision. Despite the lack of a standardized process to evaluate the adequacy of tissue samples, a definitive diagnosis was obtained in 98% (46/47). Significant complication rates (8.5%; 4/47) were consistent with those in published literature. Complications were unrelated to pre- or post-biopsy systolic blood pressure (mean 128 mmHg ±7.4, *p* = 0.25; mean 127.1 mmHg ±18.2, *p* = 0.62), platelet count (mean 295.4 PLT/µL ± 114, *p* = 0.45) or INR (mean 0.9 ± 0.1, *p* = 0.21).

## Discussion and conclusion

Our developing biopsy program continues to face challenges. The assignment of dedicated schedulers and scheduling responsibilities for biopsy clinic remains in progress. Standardized documentation templates, pre- and post-biopsy order sets, and a method for uploading signed consent forms and procedural imaging to the EMR have not been established or implemented. With a solitary nephrology attending certified in renal ultrasonography, our program lacks redundancy and is fully dependent on her schedule. The question of whether moderate sedation will be utilized for renal biopsies remains unanswered.

Launching a program to train nephrology fellows in native renal biopsy involves more than simply honing technical skills; it involves intricate consideration and interdepartmental involvement. At our institution, this process involved:

1. A champion who is heavily invested in the program’s success to be the initial driving force for change.
2. Strong collaboration from multiple departments including radiology, VIR, and pathology to ensure seamless teamwork and comprehensive patient care.
3. Addressing hospital costs linked to the training program including expenses for equipment acquisition, staffing, and procedural needs, while also optimizing resource allocation.
4. Effective communication and coordination with hospital administration, which was vital for garnering support, navigating regulatory protocols, and maintaining the sustainability of the training.
5. The ability to adapt to unanticipated challenges and circumstances beyond program control.
6. Paramount emphasis on ensuring patient safety.

We recognize that our efforts resulted in changes at only a local level. In sharing our experience, we aim to join the American Society of Nephrology, KidneyCon, GlomCon, and other passionate educators in emphasizing the need and broadening opportunities for nephrologists to continue to perform renal biopsies.
